# Supplementary material for: Frequency of physical activity during leisure time and variables related to pain and pain medication use in Spanish adults: A cross-sectional study
Source: PLoS One. 2024 Nov 13;19(11):e0310685. doi: 10.1371/journal.pone.0310685 (PMC11560030; doi:10.1371/journal.pone.0310685)
Supplement: S1 File — (DOCX) [file pone.0310685.s001.docx]

Additional file 1. Description of social classes based on occupational occupation.

| **CLASE I** - Directors and managers of establishments with 10 or more employees, and  professionals traditionally associated with university degrees |
| --- |
| **CLASE II** - Directors and managers of establishments with fewer than 10 employees,  professionals traditionally associated with university degrees and other technical support professionals.  technical support professionals. Athletes and artists |
| **CLASE III** - Intermediate occupations and self-employed workers |
| **CLASE IV** - Supervisors and workers in skilled technical occupations |
| **CLASE V** - Skilled workers in the primary sector and other semi-skilled workers  semi-skilled workers |
| **CLASE VI** - Unskilled workers |
