# Supplementary material for: Frequency of physical activity during leisure time and variables related to pain and pain medication use in Spanish adults: A cross-sectional study
Source: PLoS One. 2024 Nov 13;19(11):e0310685. doi: 10.1371/journal.pone.0310685 (PMC11560030; doi:10.1371/journal.pone.0310685)
Supplement: S2 File — (DOCX) [file pone.0310685.s002.docx]

| Additional file 2. Descriptive analysis of the Spanish population of the European Health Survey in Spain 2014. | | | | | | | |
| --- | --- | --- | --- | --- | --- | --- | --- |
| **Variables** |  | | | | | | |
| **Age (Years)** | **Men=9,535** | **Women=10,578** | **Total=20,113** | **X^2^** | **df** | **p M-W** | **V** |
| Median (IQR) | 48 (23) | 50 (25) | 49 (24) |  |  | <0.001 |  |
| **PAF** | **n (%)** | **n (%)** | **n (%)** |  |  | **p X^2^** |  |
| Inactive | 2882 (30.2) | 4062 (38.2)* | 6944 (34.5) | 334.0 | 3 | <0.001 | 0.128 |
| Occasional | 3812 (40.0) | 4480 (42.4)* | 8292 (41.2) |  |  |  |  |
| Active | 1404 (14.7) | 1008 (9.5)* | 2412 (12.0) |  |  |  |  |
| Very Active | 1437 (15.1) | 1028 (9.7)* | 2465 (12.3) |  |  |  |  |
| **Pain** | **n=9533** | **n=10572** | **Total=20105** |  |  |  | **Φ** |
| No | 6035 (63.3) | 4965 (47.0)* | 11000 (54.7) | 540.3 | 1 | <0.001 | 0.164 |
| Yes | 3498 (36.7) | 5607 (53.0)* | 9105 (45.3) |  |  |  |  |
| **Pain Level** |  |  |  |  |  |  | **V** |
| None | 6035 (63.3) | 4965 (47.0)* | 11000 (54.7) | 660.9 | 5 | <0.001 | 0.181 |
| Very Mild | 805 (8.4) | 974 (9.2) | 1779 (8.8) |  |  |  |  |
| Mild | 1195 (12.5) | 1593 (15.1)* | 2788 (13.9) |  |  |  |  |
| Moderate | 1017 (10.7) | 1905 (18.0)* | 2922 (14.5) |  |  |  |  |
| Severe | 403 (4.2) | 979 (9.3)* | 1382 (6.9) |  |  |  |  |
| Extreme | 78 (0.8) | 156 (1.5)* | 234 (1.2) |  |  |  |  |
| **Pain Affect** | **n=9534** | **n=10575** | **Total=20109** |  |  |  | **Φ** |
| No | 7412 (77.7) | 6766 (64.0)* | 14178 (70.5) | 456.6 | 1 | <0.001 | 0.151 |
| Yes | 2122 (22.3) | 3809 (36.0)* | 5931 (29.5) |  |  |  |  |
| **Pain Affect Level** |  |  |  |  |  |  | **V** |
| Nothing | 7412 (77.7) | 6766 (64.0)* | 14178 (70.5) | 485.1 | 4 | <0.001 | 0.155 |
| A little | 1009 (10.6) | 1577 (14.9)* | 2586 (12.9) |  |  |  |  |
| Moderately | 598 (6.3) | 1091 (10.3)* | 1689 (8.4) |  |  |  |  |
| Fairly | 326 (3.4) | 775 (7.3)* | 1101 (5.5) |  |  |  |  |
| A lot | 189 (2.0) | 366 (3.5)* | 555 (2.8) |  |  |  |  |
| **Pain Medication** | **n=9530** | **n=10577** | **n=20107** |  |  |  | **Φ** |
| No | 7024 (73.7) | 5959 (56.3)* | 12963 (64.6) | 660.8 | 1 | <0.001 | 0.181 |
| Yes | 2506 (26.3) | 4618 (43.7)* | 7124 (35.4) |  |  |  |  |
| **BMI** | **n=9342** | **n=10072** | **n=19414** |  |  |  | **V** |
| Underweight | 68 (0.7) | 293 (2.9)* | 361 (1.9) | 711.4 | 3 | <0.001 | 0.191 |
| Normal | 3407 (36.5) | 5201 (51.6) | 8608 (44.3) |  |  |  |  |
| Overweight | 4205 (45.0) | 2941 (29.2)* | 7146 (36.8) |  |  |  |  |
| Obesity | 1662 (17.8) | 1637 (16.3)* | 3299 (17.0) |  |  |  |  |
| **Social Class** | **n=9432** | **n=10267** | **n=19699** |  |  |  | **V** |
| I | 1131 (12.0) | 1160 (11.3) | 2291 (11.6) | 57.5 | 5 | <0.001 | 0.054 |
| II | 779 (8.3) | 940 (9.2)* | 1719 (8.7) |  |  |  |  |
| III | 1802 (19.1) | 2055 (20.0) | 3857 (19.6) |  |  |  |  |
| IV | 1535 (16.3) | 1384 (13.5)* | 2919 (14.8) |  |  |  |  |
| V | 3042 (32.3) | 3232 (31.5) | 6274 (31.8) |  |  |  |  |
| VI | 1143 (12.1) | 1496 (14.6)* | 2639 (13.4) |  |  |  |  |
| **Civil Status** | **n=9529** | **n=10564** | **n=20093** |  |  |  | **V** |
| Single | 2831 (29.7) | 2442 (23.1)* | 5273 (26.2) | 808.9 | 4 | <0.001 | 0.201 |
| Married | 5820 (61.1) | 5788 (54.8)* | 11608 (57.8) |  |  |  |  |
| Widowed | 266 (2.8) | 1373 (13.0)* | 1639 (8.2) |  |  |  |  |
| Legally separated | 237 (2.5) | 312 (3.0)* | 549 (2.7) |  |  |  |  |
| Divorced | 375 (3.9) | 649 (6.1)* | 1024 (5.1) |  |  |  |  |
| n (number of participants); IQR (Interquartile range); % (percentage); BMI (Body mass index); X^2^ (Pearson’s Chi-square); df (Degree of freedom); p W-M (p-value from Mann-Whitney U test); V (Cramer’s V coefficient); Φ (Phi coefficient); p X^2^ (p-value from Chi-square test); I-VI (Social Class, from I (Directors and managers of establishments with 10 or more employees and professionals traditionally associated with university degrees) to VI (Unskilled workers); * (Significant differences with p<0.05 from pairwise z-test from independent proportions). | | | | | | | |
