# Supplementary material for: Frequency of physical activity during leisure time and variables related to pain and pain medication use in Spanish adults: A cross-sectional study
Source: PLoS One. 2024 Nov 13;19(11):e0310685. doi: 10.1371/journal.pone.0310685 (PMC11560030; doi:10.1371/journal.pone.0310685)
Supplement: S3 File — (DOCX) [file pone.0310685.s003.docx]

| Additional file 3. Descriptive analysis of the Spanish population of the European Health Survey in Spain 2020. | | | | | | | |
| --- | --- | --- | --- | --- | --- | --- | --- |
| **Variables** |  | | | | | | |
| **Age (Years)** | **Men=9396** | **Women=9890** | **Total=19196** | **X^2^** | **df** | **p M-W** | **V** |
| Median (IQR) | 52 (24) | 53 (25) | 52 (25) |  |  | <0.001 |  |
| **PAF** | **Men=9396** | **Women=9890** | **Total=19196** |  |  | **p X^2^** |  |
| Inactive | 2926 (31.4) | 3644 (36.8)* | 6579 (34.2) | 122.8 | 3 | <0.001 | 0.080 |
| Occasional | 3616 (38.9) | 3962 (40.1)* | 7578 (39.5) |  |  |  |  |
| Active | 1132 (12.2) | 960 (9.7)* | 2092 (10.9) |  |  |  |  |
| Very Active | 1632 (17.5) | 1324 (13.4)* | 2956 (15.4) |  |  |  |  |
| **Pain** | **n=9302** | **n=9888** | **Total=19190** |  |  |  | **Φ** |
| No | 6166 (66.3) | 5112 (51.7)* | 11278 (58.8) | 420.9 | 1 | <0.001 | 0.148 |
| Yes | 3136 (33.7) | 4776 (48.3)* | 7912 (41.2) |  |  |  |  |
| **Pain Level** |  |  |  |  |  |  | **V** |
| None | 6166 (66.3) | 5112 (51.7)* | 11278 (58.8) | 512.8 | 5 | <0.001 | 0.163 |
| Very Mild | 682 (7.3) | 789 (8.0) | 1471 (7.7) |  |  |  |  |
| Mild | 1121 (12.1) | 1497 (15.1)* | 2618 (13.6) |  |  |  |  |
| Moderate | 954 (10.3) | 1609 (16.3)* | 2563 (13.4) |  |  |  |  |
| Severe | 342 (3.7) | 764 (7.7)* | 1106 (5.8) |  |  |  |  |
| Extreme | 37 (0.4) | 117 (1.2)* | 154 (0.8) |  |  |  |  |
| **Pain Affect** | **n=9303** | **n=9887** | **n=19190** |  |  |  | **Φ** |
| No | 7228 (77.7) | 6403 (64.8)* | 13631 (71.0) | 389.7 | 1 | <0.001 | 0.142 |
| Yes | 2075 (22.3) | 3484 (35.2)* | 5559 (29.0) |  |  |  |  |
| **Pain Affect Level** |  |  |  |  |  |  | **V** |
| Nothing | 7228 (77.7) | 6403 (64.8)* | 13631 (71.0) | 418.4 | 4 | <0.001 | 0.148 |
| A little | 1005 (10.8) | 1481 (15.0)* | 2486 (13.0) |  |  |  |  |
| Moderately | 634 (6.8) | 1053 (10.7)* | 1687 (8.8) |  |  |  |  |
| Fairly | 304 (3.3) | 655 (6.6)* | 959 (5.0) |  |  |  |  |
| A lot | 132 (1.4) | 295 (3.0)* | 427 (2.2) |  |  |  |  |
| **Pain Medication** | **n=9305** | **n=9888** | **n=19193** |  |  |  | **Φ** |
| No | 7147 (76.8) | 6130 (62.0)* | 13277 (69.2) | 493.4 | 1 | <0.001 | 0.160 |
| Yes | 2158 (23.2) | 3758 (38.0)* | 5916 (30.8) |  |  |  |  |
| **BMI** | **Hombres=9042** | **Mujeres=9419** | **Total=18461** |  |  |  | **V** |
| Underweight | 59 (0.8) | 271 (2.9)* | 330 (1.8) | 658.2 | 3 | <0.001 | 0.189 |
| Normal | 3184 (35.2) | 4742 (50.3)* | 7926 (42.9) |  |  |  |  |
| Overweight | 4211 (46.6) | 2966 (31.5)* | 7177 (38.9) |  |  |  |  |
| Obesity | 1588 (17.6) | 1440 (15.3)* | 3028 (16.4) |  |  |  |  |
| **Social Class** | **n=9109** | **n=9396** | **n=18505** |  |  |  | **V** |
| I | 1038 (11.4) | 1071 (11.4) | 2109 (11.4) | 112.0 | 5 | <0.001 | 0.078 |
| II | 732 (8.0) | 804 (8.6) | 1536 (8.3) |  |  |  |  |
| III | 1720 (18.9) | 2069 (22.0)* | 3789 (20.5) |  |  |  |  |
| IV | 1498 (16.4) | 1179 (12.5)* | 2677 (14.5) |  |  |  |  |
| V | 3055 (33.5) | 2901 (30.9)* | 5956 (32.2) |  |  |  |  |
| VI | 1066 (11.7) | 1372 (14.6)* | 2438 (13.2) |  |  |  |  |
| **Civil Status** | **n=9280** | **n=9856** | **n=19236** |  |  |  | **V** |
| Single | 2911 (31.4) | 2403 (24.4)* | 5314 (27.8) | 660.7 | 4 | <0.001 | 0.186 |
| Married | 5318 (57.3) | 5180 (52.6)* | 10498 (54.9) |  |  |  |  |
| Widowed | 292 (3.1) | 1237 (12.6)* | 1529 (8.0) |  |  |  |  |
| Legally separated | 271 (2.9) | 358 (3.6)* | 629 (3.3) |  |  |  |  |
| Divorced | 488 (5.3) | 678 (6.9)* | 1166 (6.1) |  |  |  |  |
| n (number of participants); IQR (Interquartile range); % (percentage); BMI (Body mass index); X^2^ (Pearson’s Chi-square); df (Degree of freedom); p W-M (p-value from Mann-Whitney U test); V (Cramer’s V coefficient); Φ (Phi coefficient); p X^2^ (p-value from Chi-square test); I-VI (Social Class, from I (Directors and managers of establishments with 10 or more employees and professionals traditionally associated with university degrees) to VI (Unskilled workers); * (Significant differences with p<0.05 from pairwise z-test from independent proportions). | | | | | | | |
