# Supplementary material for: Frequency of physical activity during leisure time and variables related to pain and pain medication use in Spanish adults: A cross-sectional study
Source: PLoS One. 2024 Nov 13;19(11):e0310685. doi: 10.1371/journal.pone.0310685 (PMC11560030; doi:10.1371/journal.pone.0310685)
Supplement: S4 File — (DOCX) [file pone.0310685.s004.docx]

| Additional file 4. Relationship between level of physical activity and prevalence of pain in the Spanish population from the European Health Survey of Spain 2014-2020. | | | | | | | | |
| --- | --- | --- | --- | --- | --- | --- | --- | --- |
| **2014** | | | | | | | | |
|  | **Overall** | | | | | | | |
| **Pain status** | **Inactive** | **Occasional** | **Active** | **Very Active** | **X^2^** | **df** | **p-value** | **V** |
| **No** | 3261 (47.0)a | 4621 (55.7)b | 1515 (62.8)c | 1603 (65.1)c | 340.2 | 3 | <0.001 | 0.130 |
| **Yes** | 3677 (53.0)a | 3670 (44.3)b | 897 (37.2)c | 861 (34.9)c |  |  |  |  |
|  | **Men** | | | | | | | |
| **No** | 1616 (56.1)a | 2415 (63.4)b | 981 (69.9)c | 1023 (71.2)c | 129.1 | 3 | <0.001 | 0.116 |
| **Yes** | 1265 (43.9)a | 1396 (36.6)b | 423 (30.1)c | 414 (28.8)c |  |  |  |  |
|  | **Women** | | | | | | | |
| **No** | 1645 (40.5)a | 2206 (49.2)b | 534 (53.0)bc | 580 (56.5)c | 128.3 | 3 | <0.001 | 0.110 |
| **Yes** | 2412 (59.5)a | 2274 (50.8)b | 474 (47.0)bc | 447 (43.5)c |  |  |  |  |
| **2020** | | | | | | | | |
|  | **Overall** | | | | | | | |
| **Pain status** | **Inactive** | **Occasional** | **Active** | **Very Active** | **X^2^** | **df** | **p-value** | **V** |
| **No** | 3493 (53.2)a | 4427 (58.4)b | 1394 (66.6)c | 1964 (66.4)c | 90.9 | 3 | <0.001 | 0.105 |
| **Yes** | 3073 (46.8)a | 3149 (41.6)b | 698 (33.4)c | 992 (33.6)c |  |  |  |  |
|  | **Men** | | | | | | | |
| **No** | 1791 (61.3)a | 2357 (65.2)b | 825 (72.9)c | 1193 (73.1)c | 81.1 | 3 | <0.001 | 0.099 |
| **Yes** | 1133 (38.7)a | 1257 (34.8)b | 307 (27.1)c | 439 (26.9)c |  |  |  |  |
|  | **Women** | | | | | | | |
| **No** | 1702 (46.7)a | 2070 (52.2)b | 569 (59.3)c | 771 (58.2)c | 211.7 | 3 | <0.001 | 0.091 |
| **Yes** | 1940 (53.3)a | 1892 (47.8)b | 391 (40.7)c | 553 (41.8)c |  |  |  |  |
| X^2^ (Pearson’s Chi-square); df (degree freedom); V (Cramer’s V coefficient); abc (Different letters indicate significant differences between proportions with p<0.05 from pairwise z-test for independent proportions). | | | | | | | | |
