# Supplementary material for: Frequency of physical activity during leisure time and variables related to pain and pain medication use in Spanish adults: A cross-sectional study
Source: PLoS One. 2024 Nov 13;19(11):e0310685. doi: 10.1371/journal.pone.0310685 (PMC11560030; doi:10.1371/journal.pone.0310685)
Supplement: S5 File — (DOCX) [file pone.0310685.s005.docx]

| Additional file 5. Relationship between level of physical activity and prevalence of pain affect in the Spanish population from the European Health Survey of Spain 2014-2020. | | | | | | | | |
| --- | --- | --- | --- | --- | --- | --- | --- | --- |
| **2014** | | | | | | | | |
|  | **Overall** | | | | | | | |
| **Pain Affect** | **Inactive** | **Occasional** | **Active** | **Very Active** | **X^2^** | **df** | **p-value** | **V** |
| **No** | 4235 (61.0)a | 6017 (72.6)b | 1919 (79.6)c | 2007 (81.4)c | 554.0 | 3 | <0.001 | 0.166 |
| **Yes** | 2706 (39.0)a | 2274 (27.4)b | 493 (20.4)c | 458 (18.6)c |  |  |  |  |
|  | **Men** | | | | | | | |
| **No** | 1994 (69.2)a | 2996 (78.6)b | 1189 (84.7)c | 1233 (85.8)c | 215.8 | 3 | <0.001 | 0.150 |
| **Yes** | 887 (30.8)a | 816 (21.4)b | 215 (15.3)c | 204 (14.2)c |  |  |  |  |
|  | **Women** | | | | | | | |
| **No** | 2241 (55.2)a | 3021 (57.4)b | 730 (72.4)c | 774 (75.3)c | 247.5 | 3 | <0.001 | 0.153 |
| **Yes** | 1819 (44.8)a | 1458 (32.6)b | 278 (27.6)c | 254 (24.7)c |  |  |  |  |
| **2020** | | | | | | | | |
|  | **Overall** | | | | | | | |
| **Pain Affect** | **Inactive** | **Occasional** | **Active** | **Very Active** | **X^2^** | **df** | **p-value** | **V** |
| **No** | 4510 (63.2)a | 5467 (72.2)b | 1657 (79.2)c | 2357 (79.7)c | 376.4 | 3 | <0.001 | 0.140 |
| **Yes** | 2415 (36.8)a | 2110 (27.8)b | 435 (20.8)c | 599 (20.3)c |  |  |  |  |
|  | **Men** | | | | | | | |
| **No** | 2082 (71.2)a | 2816 (77.9)b | 955 (84.4)c | 1375 (84.3)c | 140.7 | 3 | <0.001 | 0.123 |
| **Yes** | 842 (28.8)a | 799 (22.1)b | 177 (15.6)c | 257 (15.7)c |  |  |  |  |
|  | **Women** | | | | | | | |
| **No** | 2068 (56.8)a | 2651 (66.9)b | 702 (73.1)c | 982 (74.2)c | 190.0 | 3 | <0.001 | 0.139 |
| **Yes** | 1573 (43.2)a | 1311 (33.1)b | 258 (26.9)c | 342 (25.8)c |  |  |  |  |
| X^2^ (Pearson’s Chi-square); df (degree freedom); V (Cramer’s V coefficient); abc (Different letters indicate significant differences between proportions with p<0.05 from pairwise z-test for independent proportions). | | | | | | | | |
