# Supplementary material for: Frequency of physical activity during leisure time and variables related to pain and pain medication use in Spanish adults: A cross-sectional study
Source: PLoS One. 2024 Nov 13;19(11):e0310685. doi: 10.1371/journal.pone.0310685 (PMC11560030; doi:10.1371/journal.pone.0310685)
Supplement: S6 File — (DOCX) [file pone.0310685.s006.docx]

| Addiotional file 6. Relationship between level of physical activity and prevalence of pain medication use in the Spanish population from the European Health Survey of Spain 2014-2020. | | | | | | | | |
| --- | --- | --- | --- | --- | --- | --- | --- | --- |
| **2014** | | | | | | | | |
|  | **Overall** | | | | | | | |
| **Pain Medication** | **Inactive** | **Occasional** | **Active** | **Very Active** | **X^2^** | **df** | **p-value** | **V** |
| **No** | 4020 (57.9)a | 5492 (66.2)b | 1724 (71.5)c | 1747 (76.5)c | 237.5 | 3 | <0.001 | 0.109 |
| **Yes** | 2920 (42.1)a | 2799 (33.8)b | 688 (28.5)c | 717 (29.1)c |  |  |  |  |
|  | **Men** | | | | | | | |
| **No** | 1998 (69.4)a | 2834 (74.4)b | 1093 (77.8)b | 1099 (76.5)b | 46.8 | 3 | <0.001 | 0.070 |
| **Yes** | 881 (30.6)a | 977 (25.6)b | 311 (22.2)b | 337 (23.5)b |  |  |  |  |
|  | **Women** | | | | | | | |
| **No** | 2022 (49.8)a | 2658 (59.3)b | 631 (62.6)b | 648 (63.0)b | 121.9 | 3 | <0.001 | 0.107 |
| **Yes** | 2039 (50.2)a | 1822 (40.7)b | 377 (37.4)b | 380 (37.0)b |  |  |  |  |
| **2020** | | | | | | | | |
|  | **Overall** | | | | | | | |
| **Pain Medication** | **Inactive** | **Occasional** | **Active** | **Very Active** | **X^2^** | **df** | **p-value** | **V** |
| **No** | 4156 (63.3)a | 5271 (69.6)b | 1616 (77.2)c | 223 (24.4)c | 228.8 | 3 | <0.001 | 0.109 |
| **Yes** | 2413 (36.7)a | 2305 (30.4)b | 476 (22.8)c | 722 (24.4)c |  |  |  |  |
|  | **Men** | | | | | | | |
| **No** | 2135 (73.0)a | 2762 (76.4)b | 927 (81.9)c | 1323 (81.1)c | 57.6 | 3 | <0.001 | 0.079 |
| **Yes** | 791 (27.0)a | 853 (23.6)b | 205 (18.1)c | 309 (18.9)c |  |  |  |  |
|  | **Women** | | | | | | | |
| **No** | 2021 (55.5)a | 2509 (63.3)b | 689 (71.8)c | 911 (68.8)c | 133.8 | 3 | <0.001 | 0.116 |
| **Yes** | 1622 (44.5)a | 1452 (36.7)b | 271 (28.2)c | 413 (31.2)c |  |  |  |  |
| X^2^ (Pearson’s Chi-square); df (degree freedom); V (Cramer’s V coefficient); abc (Different letters indicate significant differences between proportions with p<0.05 from pairwise z-test for independent proportions).. | | | | | | | | |
