# Supplementary material for: Frequency of physical activity during leisure time and variables related to pain and pain medication use in Spanish adults: A cross-sectional study
Source: PLoS One. 2024 Nov 13;19(11):e0310685. doi: 10.1371/journal.pone.0310685 (PMC11560030; doi:10.1371/journal.pone.0310685)
Supplement: Attachment — Submitted filename: review.docx [file pone.0310685.s008.docx]

The manuscript examines the how physical activity can influence pain. I find the paper well-written, and it has the potential to contribute to the existing literature. However, I have some comments I would like the authors to consider.

INTRODUCTION:

The present study is conducted in the Spanish population. In line 64 to 66, authors relate high treatment costs and the access to treatment with social classes, but in Spain there is a public health system that covers the major part of the costs and is accessible for the whole population.

MATERIAL AND METHODS:

Why do the authors conduct the study with data from interviews of two different years?

In line 109 authors refers to the methodology of the surveys, could you add the reference where that methodology can be find?

The exclusion and inclusion criteria are explained in the participants section, but in the flowchart depicted in Figure 1 there are more exclusion criteria that are not explained in the participants section. In my opinion, all the exclusion criteria should be explained in that section.

In line 132-133 it is written: *Overweight (BMI between 25 and <30), and Obesity (BMI >30).* In which group are included those with BMI=30?

It should be explained that the variable pain medication is a combination of two items and that this variable is not an item of the Interview. It is confusing that the authors just consider “created variables” Pain Status and Pain affect, while pain medication is also created by the authors.

In my opinion, line 212 should be written as: “the significance level for the analyses was established at 0.05” as the significance level is a number, not an interval.

RESULTS:

A lot of information of the results is shown in additional files, some relevant results could be move to the main manuscript.

Could you please specify the p value? Writing p<0.05 does not bring much more information than that the result is significant.

Authors wrote the OR value, but they do not interpret the meaning of that value. That information is important and it should be explained in the results. Furthermore, when explaining the results, authors wrote: “*being female increases the odds of pain (OR:1.973; 95 IC 1.856-2.098) and (OR: 1.866; 95 IC 1.751-1.989)* ***times***” What does that “times” mean? This is one example, but it is repeated in several sentences.

TABLES AND FIGURES:

Figures are clear and help to read and follow the information given in the manuscript. In Figure 1 y-axes represent different numbers, some of them represent from 0 to 55 and others from 0 to 45.

In Table 1 It is written: “The significance level set at p<0.001” but in the Material and Methods section it says that the significance level is set at 0.05.

There is a mistake in Additional file 6, It is written “Addiotional”

In some Additional files differences that are significant are highlighted with abc, Why do you use different letters? Is there any difference between cells highlighted with a, with b or with c? Furthermore, in other files the significant cells are highlighted with *. I would suggest to the authors that they unify criteria.

DISCUSSION:

The following sentence is unclear: *“In contrast, in our sample those who were 288 active or very active, the prevalence obtained for these variables were the lowest”* Which variables do the authors refer to?

Please clarify the following sentence (line 291): “several studies in multiple populations have shown an relations between 292 pain scores and physical activity”

Finally, in sentence 296 authors refer to “*people who were active several times a month or a week”*. People who are considered active should do exercise continuously, someone who do exercise one week and not the next, or one month and not the next month should not be considered active.

Finally, In the limitations authors explain the possible bias due to the outbreak in Spain and its possible impact in the interview, but there is a Spanish National Health Interview in 2017. Did the authors consider used that interview? Furthermore, I do not totally understand why authors used interviews from two years if the study could have been conducted with interviews from just one year as they do not make any comparison between results in the different years.
