# Supplementary material for: Frequency of physical activity during leisure time and variables related to pain and pain medication use in Spanish adults: A cross-sectional study
Source: PLoS One. 2024 Nov 13;19(11):e0310685. doi: 10.1371/journal.pone.0310685 (PMC11560030; doi:10.1371/journal.pone.0310685)
Supplement: Attachment — Submitted filename: Reviewer 2. Round 1.docx [file pone.0310685.s009.docx]

**REVIEWER 2:**

**Dear Reviewer,**

**Thank you for your review of our manuscript. We have carefully considered your comments and believe that the quality of the paper has improved after incorporating your suggestion. Below is our response to your suggestion:**

INTRODUCTION:

The present study is conducted in the Spanish population. In line 64 to 66, authors relate high treatment costs and the access to treatment with social classes, but in Spain there is a public health system that covers the major part of the costs and is accessible for the whole population.

***Author´s response:***

**Thank you for your comment. The authors have decided to remove the sentence as it does not correspond to the context of the Spanish population.**

MATERIAL AND METHODS:

Why do the authors conduct the study with data from interviews of two different years?

***Author´s response:***

**The EHISS is carried out periodically to know the health status of the Spanish population. The last two surveys carried out were the EHISS 2014 and the EHISS 2020. We believe that including data from both provides greater strength to the results of our study. This cross-sectional study demonstrates at two different times and with a larger sample how PA was related to pain in the Spanish population.**

In line 109 authors refers to the methodology of the surveys, could you add the reference where that methodology can be find?

***Author´s response:***

**Amended**

The exclusion and inclusion criteria are explained in the participants section, but in the flowchart depicted in Figure 1 there are more exclusion criteria that are not explained in the participants section. In my opinion, all the exclusion criteria should be explained in that section.

***Author´s response:***

**Thank you for your comment. We believe that we had not explained this procedure well. In the "Participants" section we explain the general inclusion and exclusion criteria. On the other hand, in the "Variables" section we explain the exclusions made for the analyzes of specific variables. The flowchart contains both procedures.**

**Participants who met the inclusion criteria were included in the study. However, if data were not presented on any of the study variables, they were only excluded from the analysis that included said variable.**

**When citing figure 1 we have added a clarification about it thanks to your contribution.**

In line 132-133 it is written: *Overweight (BMI between 25 and <30), and Obesity (BMI >30).* In which group are included those with BMI=30?

***Author´s response:***

**Amended.**

It should be explained that the variable pain medication is a combination of two items and that this variable is not an item of the Interview. It is confusing that the authors just consider “created variables” Pain Status and Pain affect, while pain medication is also created by the authors.

***Author´s response:***

**We appreciate your comment. A sub-section called created variables has been created and it has been indicated that the variable pain medication corresponds to the combination of two items.**

In my opinion, line 212 should be written as: “the significance level for the analyses was established at 0.05” as the significance level is a number, not an interval.

***Author´s response:***

**Amended.**

RESULTS:

A lot of information of the results is shown in additional files, some relevant results could be move to the main manuscript.

***Author´s response:***

**Thank you for your comment. We agree with you. Our research includes a lot of important data in the additional material. The additional material is part of the research and can be accessed by readers. We would like you to let us know which table you would like us to include within the manuscript and not as additional material.**

Could you please specify the p value? Writing p<0.05 does not bring much more information than that the result is significant.

***Author´s response:***

**Thank you for your comment. Amended.**

Authors wrote the OR value, but they do not interpret the meaning of that value. That information is important and it should be explained in the results. Furthermore, when explaining the results, authors wrote: “*being female increases the odds of pain (OR:1.973; 95 IC 1.856-2.098) and (OR: 1.866; 95 IC 1.751-1.989)* ***times***” What does that “times” mean? This is one example, but it is repeated in several sentences.

***Author´s response:***

**Thank you for your comment. That paragraph has been rewritten so that it can be better understood.**

TABLES AND FIGURES:

Figures are clear and help to read and follow the information given in the manuscript. In Figure 1 y-axes represent different numbers, some of them represent from 0 to 55 and others from 0 to 45.

***Author´s response:***

**Amended.**

In Table 1 It is written: “The significance level set at p<0.001” but in the Material and Methods section it says that the significance level is set at 0.05.

***Author´s response:***

**That was the p value with the Bomferroni correction factor.**

There is a mistake in Additional file 6, It is written “Addiotional”

***Author´s response:***

**Amended.**

In some Additional files differences that are significant are highlighted with abc, Why do you use different letters? Is there any difference between cells highlighted with a, with b or with c? Furthermore, in other files the significant cells are highlighted with *. I would suggest to the authors that they unify criteria.

***Author´s response:***

**You refer to the results of the post hoc pairwise z test for independent proportions. It is not possible to unify criteria.**

*** was used when comparisons were made between two groups: Men and Women. If differences are found between the proportions of men and women, it is indicated with an *.**

**When comparisons were made between PAF groups, “abc” was used. Different letters indicated significant differences between PAF groups. A proportion "a" differs significantly from a proportion "b" and both differ from a proportion "c". On the other hand, if "a", "ab", "b" and "c" appear, the proportion "a" differs significantly from the proportion "b" and "c", and the "b" differs from the "c ". The proportion "bc" differs significantly from the proportion "a", but it does not differ significantly from the proportions "b" and "c".**

**For example:**

DISCUSSION:

The following sentence is unclear: *“In contrast, in our sample those who were 288 active or very active, the prevalence obtained for these variables were the lowest”* Which variables do the authors refer to?

***Author´s response:***

**We appreciate your feedback. The variables have been re-specified in this sentence to avoid confusion.**

Please clarify the following sentence (line 291): “several studies in multiple populations have shown an relations between 292 pain scores and physical activity”

***Author´s response:***

**Following your suggestion, the sentence has been rephrased to clarify the relationship between variables.**

Finally, in sentence 296 authors refer to “*people who were active several times a month or a week”*. People who are considered active should do exercise continuously, someone who do exercise one week and not the next, or one month and not the next month should not be considered active.

***Author´s response:***

**Amended. *“people who* performed physical activity *several times a month or a week”.***

Finally, In the limitations authors explain the possible bias due to the outbreak in Spain and its possible impact in the interview, but there is a Spanish National Health Interview in 2017. Did the authors consider used that interview? Furthermore, I do not totally understand why authors used interviews from two years if the study could have been conducted with interviews from just one year as they do not make any comparison between results in the different years.

***Author´s response:***

**The National Health Survey (ENS) and the European Health Survey (EHS) are different surveys. The ENS is a survey carried out at the national level promoted by the Spanish Ministry of Health. The EHS is a survey promoted by the European Statistical Office (Eurostat). We use data from the last two EHS carried out in Spain.**

**Our objective was not to compare between years, but to analyze the relationships existing at both times. We believe that testing the same hypotheses at two different points in time and with a larger population provides greater strength to our meetings, which is why we use data from the two surveys. We do not include the ENS data as it is a different survey with a different methodology.**
