# Supplementary material for: Frequency of physical activity during leisure time and variables related to pain and pain medication use in Spanish adults: A cross-sectional study
Source: PLoS One. 2024 Nov 13;19(11):e0310685. doi: 10.1371/journal.pone.0310685 (PMC11560030; doi:10.1371/journal.pone.0310685)
Supplement: Attachment — Submitted filename: Response to Reviewers.docx [file pone.0310685.s010.docx]

**EDITOR**

Dear Editor,

Thank you for considering that our article has the potential to be published in your journal. We appreciate the opportunity you have given us to improve our manuscript. We also thank the reviewers for all the comments for improvement.

Links to the primary source of data for this study are included in the "Availability of data and materials" section. This contains the complete original databases, which we believe is the most transparent way possible to replicate our study. Any researcher can follow our steps from the original databases and reach our results.

However, the final data from our research will be made available upon consistent request to the corresponding author and we have indicated this in the manuscript.

Attached are the responses to the reviewers.

Best Regards

**REVIEWER 1**

**Dear Reviewer,**

**Thank you for your review of our manuscript. We have carefully considered your comments and believe that the quality of the paper has improved after incorporating your suggestion. Below is our response to your suggestion:**

Theoretical Level:

1. Clarity and Depth in Theoretical Framework:

o The article presents a solid theoretical framework but lacks a more explicit connection between the theories presented and their application in the study.

***Author´s response:***

**Thank you for your comment. The introduction now includes a paragraph that explicitly connects the scientific evidence provided with the main objective of the study and its application.**

o It is suggested that the theoretical discussion be expanded to highlight how existing theories relate directly to the research in question. This will help strengthen the conceptual basis of the study and give coherence to the analysis.

***Author´s response:***

**Thank you very much for your contribution. The discussion section has been expanded, clearly improving the quality of the research as well as reinforcing the coherence of the explored relationship.**

Methodological Level:

2. Details and Clarity of Methodology:

o The methodological description is fairly concise and could be more detailed to allow for accurate replication of the study by other researchers.

It is recommended that the methodological section be expanded to include more information about the study design, sample selection, instruments used and specific procedures. This will increase the reliability and validity of the results.

***Author´s response:***

**Thank you for your comment.**

**Although we had previously included them in the data accessibility section. After your comment we have included in the methodology the links to the databases of our study. From them you will be able to download the database in different formats and you will be able to follow step by step the procedures explained in our manuscript and that we performed in our research.**

**References 22 and 23 included the survey methodologies, explaining in detail the sample calculation, the sampling system, how missing data were treated and other detailed information about the procedures performed. We included the information necessary for our study to be replicated, but we did not go into the survey methodology in detail so as not to commit plagiarism and not to include information that has already been detailed in the bibliography. If you think we should include any specific information, we would like you to let us know so that we can improve the comprehensibility of our manuscript.**

**The data are public and open access, which makes it easier for our research to be replicated by any researcher. However, we have made minor changes to this section to improve understanding.**

Bibliographical references:

3. Updating and Extending References:

o Bibliographic references are adequate but could benefit from more diversity and updating, especially in relation to the most recent developments in the field.

***Author´s response:***

**We appreciate your comment. The bibliographical references are adequate and have been fully updated as well as related to more recent developments and research in this field of knowledge.**

o Seek out and add new and recent research sources that support and enrich the arguments presented. This will strengthen the credibility of the article by demonstrating a thorough understanding of the current state of the field.

***Author´s response:*** Thank you for your comment. Following your suggestion, new research has been added to enrich the arguments presented in the article.

**REVIEWER 2:**

**Dear Reviewer,**

**Thank you for your review of our manuscript. We have carefully considered your comments and believe that the quality of the paper has improved after incorporating your suggestion. Below is our response to your suggestion:**

INTRODUCTION:

The present study is conducted in the Spanish population. In line 64 to 66, authors relate high treatment costs and the access to treatment with social classes, but in Spain there is a public health system that covers the major part of the costs and is accessible for the whole population.

***Author´s response:***

**Thank you for your comment. The authors have decided to remove the sentence as it does not correspond to the context of the Spanish population.**

MATERIAL AND METHODS:

Why do the authors conduct the study with data from interviews of two different years?

***Author´s response:***

**The EHISS is carried out periodically to know the health status of the Spanish population. The last two surveys carried out were the EHISS 2014 and the EHISS 2020. We believe that including data from both provides greater strength to the results of our study. This cross-sectional study demonstrates at two different times and with a larger sample how PA was related to pain in the Spanish population.**

In line 109 authors refers to the methodology of the surveys, could you add the reference where that methodology can be find?

***Author´s response:***

**Amended**

The exclusion and inclusion criteria are explained in the participants section, but in the flowchart depicted in Figure 1 there are more exclusion criteria that are not explained in the participants section. In my opinion, all the exclusion criteria should be explained in that section.

***Author´s response:***

**Thank you for your comment. We believe that we had not explained this procedure well. In the "Participants" section we explain the general inclusion and exclusion criteria. On the other hand, in the "Variables" section we explain the exclusions made for the analyzes of specific variables. The flowchart contains both procedures.**

**Participants who met the inclusion criteria were included in the study. However, if data were not presented on any of the study variables, they were only excluded from the analysis that included said variable.**

**When citing figure 1 we have added a clarification about it thanks to your contribution.**

In line 132-133 it is written: *Overweight (BMI between 25 and <30), and Obesity (BMI >30).* In which group are included those with BMI=30?

***Author´s response:***

**Amended.**

It should be explained that the variable pain medication is a combination of two items and that this variable is not an item of the Interview. It is confusing that the authors just consider “created variables” Pain Status and Pain affect, while pain medication is also created by the authors.

***Author´s response:***

**We appreciate your comment. A sub-section called created variables has been created and it has been indicated that the variable pain medication corresponds to the combination of two items.**

In my opinion, line 212 should be written as: “the significance level for the analyses was established at 0.05” as the significance level is a number, not an interval.

***Author´s response:***

**Amended.**

RESULTS:

A lot of information of the results is shown in additional files, some relevant results could be move to the main manuscript.

***Author´s response:***

**Thank you for your comment. We agree with you. Our research includes a lot of important data in the additional material. The additional material is part of the research and can be accessed by readers. We would like you to let us know which table you would like us to include within the manuscript and not as additional material.**

Could you please specify the p value? Writing p<0.05 does not bring much more information than that the result is significant.

***Author´s response:***

**Thank you for your comment. Amended.**

Authors wrote the OR value, but they do not interpret the meaning of that value. That information is important and it should be explained in the results. Furthermore, when explaining the results, authors wrote: “*being female increases the odds of pain (OR:1.973; 95 IC 1.856-2.098) and (OR: 1.866; 95 IC 1.751-1.989)* ***times***” What does that “times” mean? This is one example, but it is repeated in several sentences.

***Author´s response:***

**Thank you for your comment. That paragraph has been rewritten so that it can be better understood.**

TABLES AND FIGURES:

Figures are clear and help to read and follow the information given in the manuscript. In Figure 1 y-axes represent different numbers, some of them represent from 0 to 55 and others from 0 to 45.

***Author´s response:***

**Amended.**

In Table 1 It is written: “The significance level set at p<0.001” but in the Material and Methods section it says that the significance level is set at 0.05.

***Author´s response:***

**That was the p value with the Bomferroni correction factor.**

There is a mistake in Additional file 6, It is written “Addiotional”

***Author´s response:***

**Amended.**

In some Additional files differences that are significant are highlighted with abc, Why do you use different letters? Is there any difference between cells highlighted with a, with b or with c? Furthermore, in other files the significant cells are highlighted with *. I would suggest to the authors that they unify criteria.

***Author´s response:***

**You refer to the results of the post hoc pairwise z test for independent proportions. It is not possible to unify criteria.**

*** was used when comparisons were made between two groups: Men and Women. If differences are found between the proportions of men and women, it is indicated with an *.**

**When comparisons were made between PAF groups, “abc” was used. Different letters indicated significant differences between PAF groups. A proportion "a" differs significantly from a proportion "b" and both differ from a proportion "c". On the other hand, if "a", "ab", "b" and "c" appear, the proportion "a" differs significantly from the proportion "b" and "c", and the "b" differs from the "c ". The proportion "bc" differs significantly from the proportion "a", but it does not differ significantly from the proportions "b" and "c".**

**For example:**

DISCUSSION:

The following sentence is unclear: *“In contrast, in our sample those who were 288 active or very active, the prevalence obtained for these variables were the lowest”* Which variables do the authors refer to?

***Author´s response:***

**We appreciate your feedback. The variables have been re-specified in this sentence to avoid confusion.**

Please clarify the following sentence (line 291): “several studies in multiple populations have shown an relations between 292 pain scores and physical activity”

***Author´s response:***

**Following your suggestion, the sentence has been rephrased to clarify the relationship between variables.**

Finally, in sentence 296 authors refer to “*people who were active several times a month or a week”*. People who are considered active should do exercise continuously, someone who do exercise one week and not the next, or one month and not the next month should not be considered active.

***Author´s response:***

**Amended. *“people who* performed physical activity *several times a month or a week”.***

Finally, In the limitations authors explain the possible bias due to the outbreak in Spain and its possible impact in the interview, but there is a Spanish National Health Interview in 2017. Did the authors consider used that interview? Furthermore, I do not totally understand why authors used interviews from two years if the study could have been conducted with interviews from just one year as they do not make any comparison between results in the different years.

***Author´s response:***

**The National Health Survey (ENS) and the European Health Survey (EHS) are different surveys. The ENS is a survey carried out at the national level promoted by the Spanish Ministry of Health. The EHS is a survey promoted by the European Statistical Office (Eurostat). We use data from the last two EHS carried out in Spain.**

**Our objective was not to compare between years, but to analyze the relationships existing at both times. We believe that testing the same hypotheses at two different points in time and with a larger population provides greater strength to our meetings, which is why we use data from the two surveys. We do not include the ENS data as it is a different survey with a different methodology.**
