# Supplementary material for: Frequency of physical activity during leisure time and variables related to pain and pain medication use in Spanish adults: A cross-sectional study
Source: PLoS One. 2024 Nov 13;19(11):e0310685. doi: 10.1371/journal.pone.0310685 (PMC11560030; doi:10.1371/journal.pone.0310685)
Supplement: Attachment — Submitted filename: reviewII.docx [file pone.0310685.s011.docx]

Thank you to the authors for amended my previous comments and recommendations and congratulations for your work.

There is just one comment that, from my point of view, that it is not completely solved. It is the one related to the OR and the word “times” when authors describe the results: It does not make sense to put the OR and 95CI between brackets and the word times out of brackets, when this word refers to that information. I think authors should dropped the word times from there.

Regarding the table that can be moved from the additional files to the manuscript, I think authors have to choose the table with more relevant and compelling information for the study.
