# Supplementary material for: Frequency of physical activity during leisure time and variables related to pain and pain medication use in Spanish adults: A cross-sectional study
Source: PLoS One. 2024 Nov 13;19(11):e0310685. doi: 10.1371/journal.pone.0310685 (PMC11560030; doi:10.1371/journal.pone.0310685)
Supplement: Attachment — Submitted filename: Response to Reviewers.docx [file pone.0310685.s012.docx]

**Dear Reviewer 2,**

**Thank you for your comment and for helping us to improve our manuscript, we have made further modifications following your recommendations.**

Thank you to the authors for amended my previous comments and recommendations and congratulations for your work.

There is just one comment that, from my point of view, that it is not completely solved. It is the one related to the OR and the word “times” when authors describe the results: It does not make sense to put the OR and 95CI between brackets and the word times out of brackets, when this word refers to that information. I think authors should dropped the word times from there.

**Author’s Response:**

**Thank you for your comment. There was indeed an error in the wording of the results of the regression models and these have been amended.**

Regarding the table that can be moved from the additional files to the manuscript, I think authors have to choose the table with more relevant and compelling information for the study.

**Author’s Response:**

**Regarding the tables included in the supplementary material.**

**After considering the structure of the article, we decided to unify additional tables 7 and 8. These tables showed the results of multiple logistic regression analyses on pain, affected by pain and the use of pain medication.**

**As these tables are extensive, the authors have simplified and merged them into the current table 2 in the main manuscript.**

**We have decided on these tables as we believe they provide relevant data that complement the wording of the results and help readers to understand the results.**

**The rest of the tables continue in the additional files but in the main manuscript are the figures showing the data from these tables.**

**The reader who wants to go deeper into them will be able to access the additional material and find what he/she needs. The additional material is part of the research and is accessible to all, we believe that this does not compromise the understanding of the research.**

**Best Regards**
