# Supplementary material for: Frequency of physical activity during leisure time and variables related to pain and pain medication use in Spanish adults: A cross-sectional study
Source: PLoS One. 2024 Nov 13;19(11):e0310685. doi: 10.1371/journal.pone.0310685 (PMC11560030; doi:10.1371/journal.pone.0310685)
Supplement: Attachment — Submitted filename: Rebuttal Letter Final Round.docx [file pone.0310685.s013.docx]

Dear Editor,

The co-authors welcome all your comments and recommendations to improve the presentation of our research.

As you have suggested, we have made all the final adjustments noted.

*“Your title is long and not very intuitive. I suggest you change it to “Frequency of physical activity during leisure time and its effect on pain variables and medication use: A Spanish Cross-Sectional Study”. Think about whether this makes sense for your study (it is just a suggestion)”.*

**Author’s response:**

According to your suggestions, we have modified the title to make it shorter. However, we believe that we should not talk about ‘effects’ as this is a cross-sectional study. Therefore, we have not included this term in the title.

*“Organize the abstract into topics (Background [research question]; Objective; Methods; Results; Conclusion)”.*

**Author’s response:**

We have made the modifications you suggested.

*“The objective stated in your abstract is different from the objective stated in your introduction. Please correct this. The sentence describing the objective must be exactly the same in both”.*

**Author’s response:**

Amended.

*“In the methods section, include a topic called "Sample Size" and explain in detail the sampling strategies used in this study”.*

**Author’s response:**

All information on this can be found in the survey methodology. This was indicated in the methodology section. We have added a new sub-section so that it can be better located.

*“In the conclusion, objectively describe your final considerations in a single paragraph. Avoid phrases like “Based on the results of this study” or “In our sample the results of this study”. The conclusion described in the abstract and the conclusion described after the discussion must be exactly the same”.*

**Author’s response:**

Thank you for your comment. The conclusions have been rewritten.
